# Correction: Chiral ruthenium polypyridyl complexes as mitochondria-targeted apoptosis inducers

**DOI:** 10.1039/c8md90010h

**Published:** 2018-03-19

**Authors:** Tianfeng Chen, Wen-Jie Mei, Yum-Shing Wong, Jie Liu, Yanan Liu, Huang-Song Xie, Wen-Jie Zheng

**Affiliations:** a Department of Chemistry , Jinan University , Guangzhou , 510632 , China . Email: tzhwj@jnu.edu.cn ; Fax: +86 20 8522 1263 ; Tel: +86 20 8522 1263; b School of Chemistry and Chemical Engineering , Guangdong Pharmaceutical University , Guangzhou , P.R. China . Email: wenjiemei@126.com ; Fax: +86 20 8522 1263 ; Tel: +86 20 8522 1263; c Department of Biology , The Chinese University of Hong Kong , Hong Kong , SAR , China . Email: yumshingwong@cuhk.edu.hk ; Fax: +852 2603 5745 ; Tel: +852 2609 6389

## Abstract

Correction for ‘Chiral ruthenium polypyridyl complexes as mitochondria-targeted apoptosis inducers’ by Tianfeng Chen *et al.*, *Med. Chem. Commun.*, 2010, **1**, 73–75.



## 

The authors regret that in Table 1 of their manuscript the data for **Δ-3** and **Λ-3** were swapped. The corrected table is shown below.

**Table 1:** Cytotoxic effects of chiral Ru polypyridyl complexes on human cancer and normal cell lines

IC_50_/μM						
Complexes	A375	HepG2	SW620	PC-3	HS68	HK-2
**Δ-1**	35.3 ± 4.2	19.9 ± 2.5	45.2 ± 3.3	99.1 ± 7.2	—	—
**Λ-1**	28.1 ± 3.7	11.4 ± 1.3	29.9 ± 2.0	82.7 ± 5.6	—	—
**Δ-2**	48.4 ± 6.3	16.6 ± 2.3	36.6 ± 4.5	>200	—	—
**Λ-2**	49.9 ± 8.5	19.9 ± 1.7	51.0 ± 3.4	>200	—	—
**Δ-3**	17.7 ± 2.6	54.8 ± 6.4	33.2 ± 2.2	>200	—	—
**Λ-3**	5.9 ± 1.1	10.0 ± 1.2	18.9 ± 0.9	79.5 ± 8.1	18.8 ± 3.9	108.5 ± 9.3
Cisplatin	7.3 ± 0.8	13.6 ± 2.0	30.0 ± 4.1	36.2 ± 2.9	1.8 ± 0.7	10.3 ± 2.1

In addition, on page 74, left column, top paragraph, the IC_50_ values for cisplatin against HS68 and HK-2 cells should be corrected to show ‘cisplatin (1.8 and 10.3 μM respectively)’ instead of ‘cisplatin (4.8 and 3.2 μM respectively)’.

The Royal Society of Chemistry apologises for these errors and any consequent inconvenience to authors and readers.

